# Identification and Expression Analysis of *Gretchen Hagen 3 (GH3)* in Kiwifruit (*Actinidia chinensis*) During Postharvest Process

**DOI:** 10.3390/plants8110473

**Published:** 2019-11-06

**Authors:** Zengyu Gan, Liuying Fei, Nan Shan, Yongqi Fu, Jinyin Chen

**Affiliations:** 1 Jiangxi Key Laboratory for Postharvest Technology and Nondestructive Testing of Fruits & Vegetables, Collaborative Innovation Center of Postharvest Key Technology and Quality Safety of Fruits and Vegetables, Jiangxi Agricultural University, Nanchang 330045, China; ganzy@jxau.edu.cn (Z.G.); feiliuying1111@163.com (L.F.); yqzq2009@126.com (Y.F.); 2 College of Agronomy, Jiangxi Agricultural University, Nanchang 330045, China; shanjxau@163.com; 3 Pingxiang University, Pingxiang 337055, China

**Keywords:** *GH3* gene, kiwifruit, phylogenetic analysis, gene expression, promoter activity

## Abstract

In plants, the Gretchen *GH3* (GH3) protein is involved in free auxin (IAA) and amino acid conjugation, thus controlling auxin homeostasis. To date, many *GH3* gene families have been identified from different plant species. However, the *GH3* gene family in kiwifruit (*Actinidia chinensis*) has not been reported. In this study, 12 *AcGH3* genes were identified, phylogenetic analysis of AtGH3 (*Arabidopsis*), SlGH3 (*Solanum lycopersicum*), and AcGH3 provided insights into various orthologous relationships among these proteins, which were categorized into three groups. Expression analysis of *AcGH3* genes at different postharvest stages suggested limited or no role for most of the *AcGH3* genes at the initiation of fruit ripening. *AcGH3.1* was the only gene exhibiting ripening-associated expression. Further study showed that the expression of *AcGH3.1* gene was induced by NAA (1-naphthylacetic acid, auxin analogue) and inhibited by 1-MCP (1-methylcyclopropene, ethylene receptor inhibitor), respectively. *AcGH3.1* gene silencing inhibited gene expression and delayed fruit softening in kiwifruit. The results indicate that *AcGH3.1* may play an important role in the softening process of fruits. Analysis of the *AcGH3.1* promoter revealed the presence of many cis-elements related to hormones, light, and drought. The determination of GUS (β-Galactosidase) enzyme activity revealed that promoter activity increased strikingly upon abscisic acid (ABA), ethylene, or NAA treatment, and significantly decreased with salicylic acid (SA) treatment. The present study could help in the identification of *GH3* genes and revelation of *AcGH3.1* gene function during postharvest stages, which pave the way for further functional verification of the *AcGH3.1* gene.

## 1. Introduction

Kiwifruits have long been called the ‘king of fruits’ due to its unique flavor and exceptional nutritional value. However, the fruit quality of kiwifruits not only declines rapidly after ripening, but also is not resistant to storage and transportation. The speed of softening after harvesting directly determines the shelf life, flavor and edible value of the fruit. Many studies have found that auxin (IAA) plays an important role in fruit ripening and aging. Exogenous IAA treatment of immature tomato fruit can delay fruit ripening and lead to parthenocarpy [1]. It is believed that IAA can delay the ripening of the climacteric fruit, and the fruit starts to fully mature due to the loss of hormone function of IAA [2].

Reduced IAA levels in fruits entering the mature stage can be achieved and maintained through changes in certain aspects of auxin metabolism [3,4]. Among them, IAA conjugation with amino acids is of particular interest because it is catalyzed by auxin induced proteins to provide a negative feedback loop to control auxin homeostasis [5]. Currently, various IAA-amino acid conjugates have been identified as endogenous compounds in a range of different plant species. In fruits such as tomatoes, grapes, pears, etc., the content of free IAA began to decrease before fruit ripening, which was accompanied by the increase of conjugated IAA (IAA-Asp) level, and it was believed that the ratio of free IAA/conjugated IAA determined the ripening of fruits, rather than the absolute amount of free IAA [1,6,7]. The conjugating reaction of free IAA to conjugated IAA is catalyzed by IAA amino acid synthase, which is encoded by the *Gretchen Hagen 3 (GH3)* gene. These proteins negatively regulate auxin homeostasis in plants [8]. Overexpression of *OsGH3.1* resulted in reduced free IAA content and dwarfing of transgenic rice plants [9]. In addition, the identification of many IAA-amino acid conjugates as endogenous compounds in other different plant species has confirmed this mechanism in plants [10,11]. The first *GH3* gene was isolated from soybean by differential screening after auxin treatment, and its transcription level was induced within 10 min after auxin treatment [8]. Since then, several members of the gene family have been identified in many plant species ranging from moss to gymnosperm to angiosperms [12,13,14]. 

Recent studies have shown that GH3 protein is involved in the growth and development of fleshy fruit, mainly by regulating the level of free IAA in the fruit to regulate fruit development and maturity. The role of the mature-associated GH3-1 protein in regulating endogenous IAA concentrations was investigated in grape berries and it was observed that this protein may be involved in establishing and maintaining low IAA concentrations in mature berries [15]. Further studies revealed that another GH3 protein, GH3-2, is also involved in the fruit ripening of grape berries [6]. In addition, differential expressions of two *GH3* genes have also been reported during the development of the non-climacteric fruit longan. Among them, *DlGH3.1* gene expression might be associated with pericarp growth, while *DlGH3.2* accumulation is likely to be related to both pericarp growth and fruit ripening [16]. A *CcGH3* gene was isolated from pepper, which was highly expressed in pericarp and placenta, and over-expressed in tomato accelerated fruit ripening, indicating that the *CcGH3* gene was involved in fruit ripening [17]. Fifteen *GH3* gene family members were isolated in a tomato, in which *SlGH3-1* and *SlGH3-2* showed mature-related expression and induction of auxin and ethylene. Therefore, this gene can be used to manipulate the maturation process in tomato fruit [18].

Kiwifruits are a climacteric fruit that softens quickly in the short term after harvest, which has huge limitations on its market value. IAA inhibits the ripening of kiwifruit, but the mechanism is not clear. Our study provides comprehensive information on the *GH3* gene from kiwifruit, and analyzed the expression patterns during the postharvest process. We also analyzed the promoter activity of the *AcGH3.1* gene and laid the foundation for studying its upstream regulation.

## 2. Results

### 2.1. Identification and Phylogenetic Analysis of AcGH3 Family Genes in Kiwifruit

To identify the *GH3* family genes in kiwifruit, BLAST searches of the kiwifruit genome database (http://bioinfo.bti.cornell.edu/cgi-bin/kiwi/blast.cgi) were performed using the GH3 of the *Arabidopsis* and tomato proteins as a query sequence. A total of 12 *GH3* genome DNA sequences were identified in *A. chinensis*; these genes were named as *AcGH3.1-AcGH3.12*. The names, IDs, open reading frame (ORF) lengths, position and deduced polypeptides of *AcGH3* gene are shown in Table 1. The open reading frame (ORF) length of the *AcGH3* gene varied from 1437 bp (*AcGH3.1*) to 2163 bp (*AcGH3.5*) and encoded 478–720aa peptides with a predicted molecular weight range of 54.29–81.26 kD. The theoretical pI ranged from 5.28 (AcGH3.4) to 8.53 (AcGH3.2) (Table 1). Except that *AcGH3.5* and *AcGH3.10* are distributed on chromosome 23, the other 10 *GH3* members are distributed on different chromosomes. In addition, the chromosomal location of *AcGH3.7* is unknown (Table 1). 

**Figure 1:**
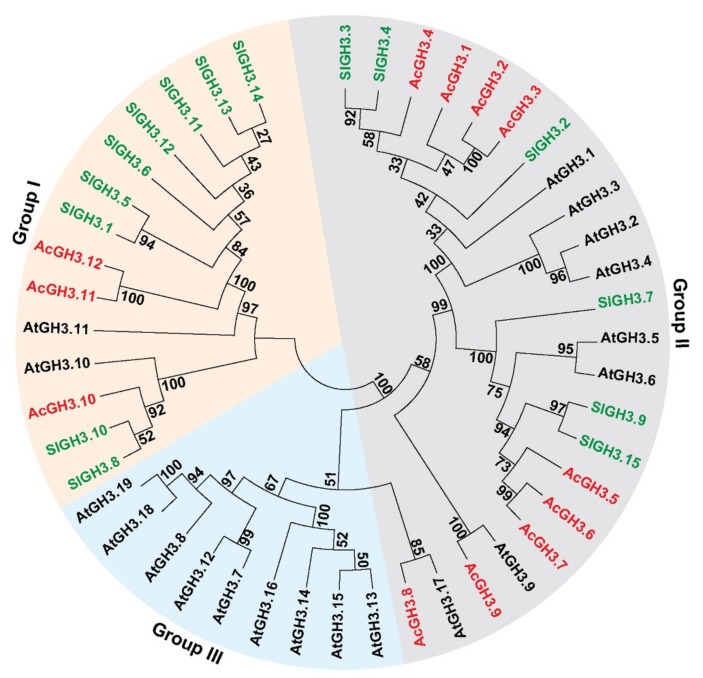
Phylogenetic tree derived from the amino-acid sequences of 12 *AcGH3* genes in kiwifruit and 15 *SlGH3* genes in tomato, and 19 *AtGH3* genes in *Arabidopsis*. All the *GH3* genes are divided into three groups (I–III), and numbers near branches represent bootstrap values. The red, green and black fonts indicate GH3s from kiwifruit, tomato, and *Arabidopsis*, respectively.

To investigate the relationship of the GH3 proteins, the full-length protein sequences of the 19 AtGH3s, 15 SlGH3s, and 12 AcGH3s were used to build the phylogenetic tree (Figure 1). The results indicated that the *GH3* gene family could be grouped into three major subfamilies, I, II, and III. AcGH3.10–AcGH3.12 were grouped into subfamily I, the similarity of the three AcGH3 proteins is 43.9–93.2%. AcGH3.1–AcGH3.9 were grouped into subfamily III, the similarity of the nine AcGH3 proteins is between 35.9–93.5% (Figure 1; Table 2). No AcGH3 belonged to subfamily III. All 12 AcGH3s were distributed into 3 sister pairs of paralogous GH3s (AcGH3.2/AcGH3.3 (93.5%), AcGH3.6/AcGH3.7 (90.2%), AcGH3.11/AcGH3.12 (93.2%)), which had very strong bootstrap support (≥99), while the remaining AcGH3 were not matched (Figure 1; Table 2).

### 2.2. Expression Analysis of AcGH3 Gene Family

**Figure 2:**
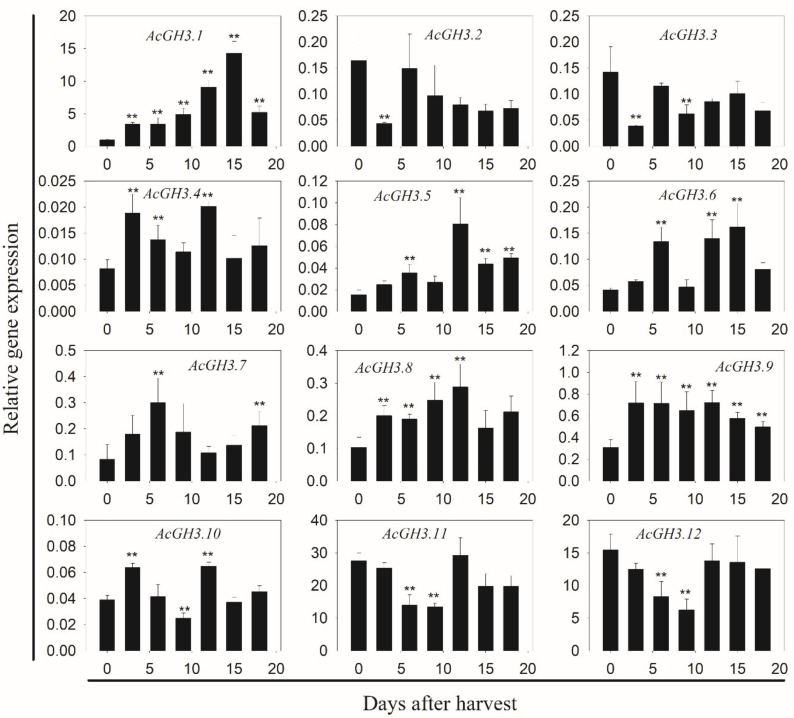
q-PCR analysis of 12 *AcGH3* genes in a total of seven postharvest stages. X-axis represents the different stages of postharvest. Y-axis represents the relative mRNA levels of genes. Expression of *Actin* was used as an internal control and to normalize the expression of *AcGH3* genes. All values in the figure are relative to the *AcGH3.1* value on day zero after harvest. Error bars show the standard error between three replicates performed. Asterisks indicate a significant difference relative to day zero after harvest according to a student’s t-test (** *p* < 0.01).

**Figure 3:**
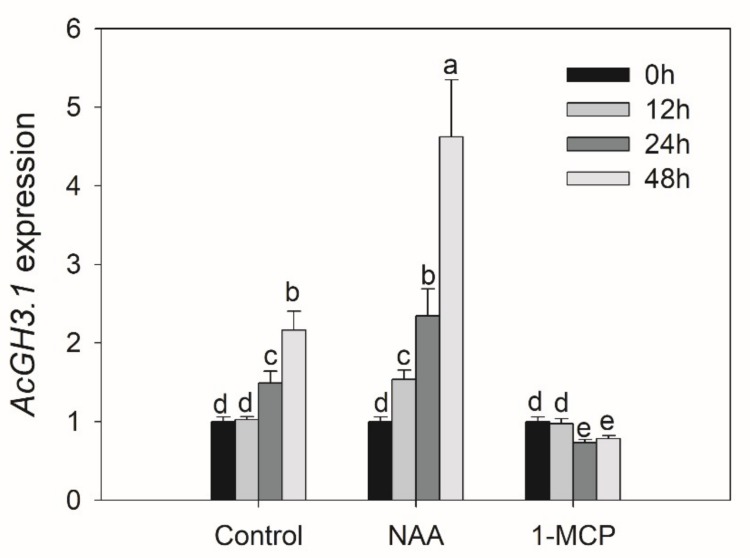
Effects of NAA and 1-MCP treatment on *AcGH3.1* gene expression in fruit after harvest. All values in the figure are relative to control value 0 h after harvest. Expression of *Actin* was used as an internal control and to normalize the expression of *AcGH3.1* gene. Error bars show the standard error between three replicates performed. Bars with the same letter are not significantly different at the 0.01 level according to a student’s t-test.

In order to clarify the function of the *AcGH3* gene after harvest, we analyzed the changes of expression levels in seven different postharvest stages. In group I, *AcGH3.10* is hardly expressed, *AcGH3.11* and *AcGH3.12* were the main expression members, the similarity between them is extremely high, and the expression trend is relatively consistent. They showed a decrease in expression level on day 0–9, followed by a rise (Figure 2). In group II, the C_T_ values of *AcGH3.4* and *AcGH3.5* > 36, which hardly expressed. While *AcGH3.2*, *AcGH3.3*, *AcGH3.6*–*AcGH3.9* showed very low expression levels in the fruit postharvest period. *AcGH3.1* was the only gene exhibiting ripening-associated expression, its expression increased significantly, peaked at 15 days, and then declined at 18 days (Figure 2). Therefore, we suspect that *AcGH3.1* may be related to the ripening process of the kiwifruit. We tested the response of *AcGH3.1* gene expression to NAA (auxin analogue) and 1-MCP (ethylene receptor inhibitor), it was found that NAA can significantly promote the expression of *AcGH3.1*, while 1-MCP inhibits the increase of its expression (Figure 3). 

### 2.3. Downregulation of the AcGH3.1 Gene Alter Fruit Maturation

**Figure 4:**
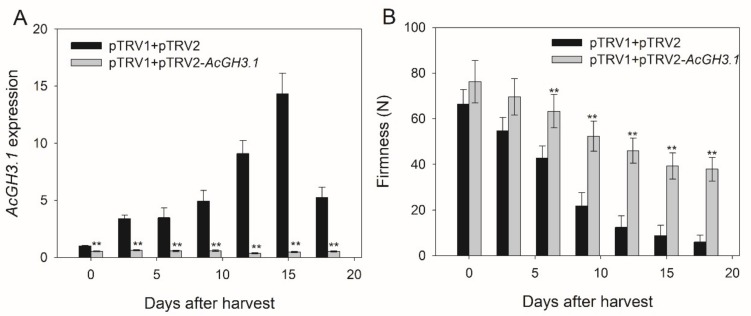
Effects of *AcGH3.1* silence on (**A**) *AcGH3.1* expression and (**B**) firmness during kiwifruit postharvest storage. Expression of *Actin* was used as an internal control and to normalize the expression of *AcGH3* genes. Error bars show the standard error between three replicates performed. Asterisks indicate a significant difference relative to pTRV1 + pTRV2 according to a student’s t-test (** *p* < 0.01).

To investigate the relationship of *AcGH3.1* with fruit softening and ripening during postharvest, we generated the *AcGH3.1*-silenced construct by cloning the specific fragment (486 bp of the ORF) into the restriction site of *Xba I* and *BamH I* in the pTRV2 vector. Next, the purified plasmids of pTRV1, pTRV2, and pTRV2-*AcGH3.1* were transformed into *A. tumefaciens* strain GV3101. The *Agrobacterium strain* was infiltrated into the kiwifruit pedicel about seven days before harvest. After this period, the fruits of pTRV1 + pTRV2-*AcGH3.1* were collected. The expression level of *AcGH3.1* was analyzed by qRT-PCR, its expression level in pTRV1+pTRV2-*AcGH3.1* fruits was lower than that in control fruit throughout the storage period, and the decrease in firmness of pTRV1 + pTRV2-*AcGH3.1* fruits was significantly inhibited (Figure 4). These results indicated that *AcGH3.1* gene silencing delayed fruit softening.

### 2.4. Analysis of Cis-Acting Elements of AcGH3.1 Gene Promoter

PlantCARE was used to predict the upstream promoter elements of the gene and analyze the number of cis-acting elements related to hormones, light, and drought to further explore the functions of *AcGH3.1*. On the promoter of *AcGH3.1*, hormone-related action elements were relatively abundant, such as ABRE (abscisic acid (ABA) responsive element), ERE-motif (ethylene response factor binding site), TCA-element (salicylic acid (SA)-responsive element.), and TGA-element (auxin-responsive element) (Table 3). These plant hormones are reportedly involved in fruit ripening and softening, and can help us to better understand the transcriptional regulation of the *AcGH3.1* gene. 

**Figure 5:**
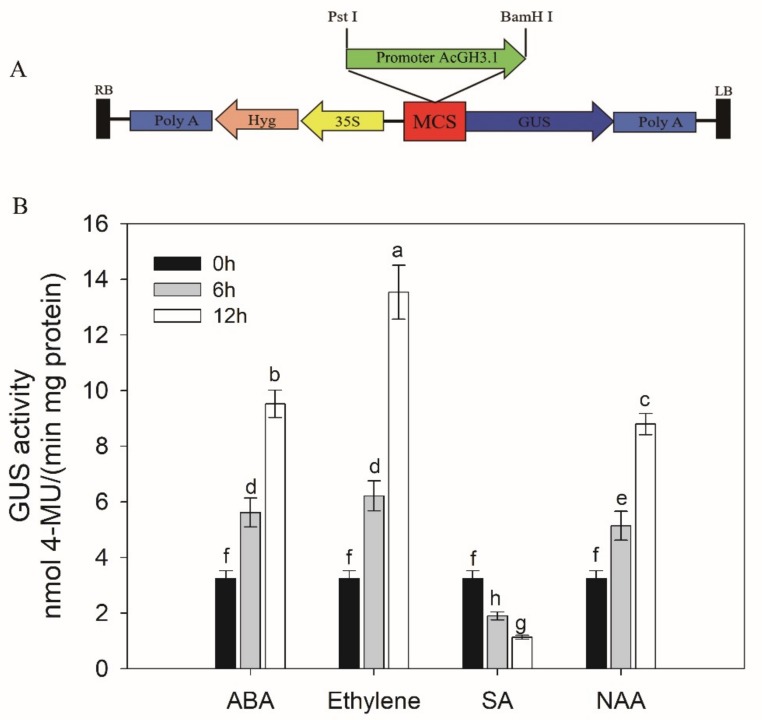
(**A**) Schematic representation of *AcGH3.1* promoter cloned in pCAMBIA1391 vector (promoter-less vector) at *Pst I* and *BamH I* sites for measuring GUS activity and field-infiltration; (**B**) Comparison of GUS activity determined in protein extracts (in vitro) treated with ABA, ethylene, SA, and NAA. Error bars show the standard error between three replicates performed. Bars with the same letter are not significantly different at the 0.01 level.

Tobacco leaves are usually used to measure promoter activity. To further verify that *AcGH3.1* expression was the above hormone inducible, fusion construct *proAcGH3.1-GUS* was transiently expressed in tobacco leaves and used to check the activities of the promoter (Figure 5A). After three days, we analyzed the effect of ABA, ethylene, SA, or NAA on *proAcGH3.1-GUS*, treated the transiently transformed tobacco leaves with ABA, ethylene, SA, or NAA, while tobacco leaves treated with H_2_O were used as controls. The determination of GUS enzyme activity revealed that GUS activity increased strikingly upon ABA, ethylene, or NAA treatment, and significantly decreased with SA treatment (Figure 5B). These observations suggested that AcGH3.1 expression is affected by ABA, ethylene, SA, or NAA, which might be closely related to fruit softening and ripening. 

## 3. Discussion

As supported by an increasing amount of data, auxin plays an important role in the initiation of fruit ripening. The conjugation of IAA to amino acids, catalyzed by GH3 proteins, is an important aspect in the control of auxin homeostasis in plants [3,4,14]. It is necessary to study the transcription and function of members of this gene family, so that the information could be used for understand the auxin metabolism during fruit development and ripening. The first *GH3* gene was isolated from soybean by differential screening after auxin treatment [8]. Nineteen *GH3* genes were identified from *Arabidopsis* and 12 from rice [5,20]. Since then, several members of the gene family have been identified in many plant species, such as, grape, orange, tomato, papaya, etc. [15,18,21,22]. Here, 12 *GH3* genes were identified from the kiwifruit genome. In the combined phylogenetic tree of *Arabidopsis*, tomato, and kiwifruit, all the GH3 proteins were found to cluster in three groups. AcGH3 is all grouped into subfamily I and II, and absence of any AcGH3 gene in group III. Presence of one sister pairing (AcGH3.9–AtGH3.9, 100%) in the combined phylogenetic tree, indicating a close relationship between the *Arabidopsis* and kiwifruit *GH3* gene families, these genes might have descended from a common ancestor and can have conserved functions [23].

Gene expression analysis indicated that the *GH3* gene has different expression patterns and function in different plants [16,24,25]. Two *Arabidopsis* GH3 proteins, DFL1 and YDK1, negatively regulate shoot bud cell elongation and lateral root formation, overexpressing *GH3-2/YDK1* and *GH3-6/DFL1* mutants showed a dwarfing phenotype consistent with these reduced levels of free IAA [26,27]. The *AtGH3-9* gene is involved in the regulation of the primary root growth of *Arabidopsis*, the overexpressed strains of *GH3-8* showed decreased IAA level and increased IAA-Asp level, abnormal development morphology and growth retardation [28]. Overexpression of a rice *GH3* gene (*GH3.13*) related to drought adaptation showed a dwarfing phenotype with increased tiller number and leaf angle [29]. The expression levels of the ancestral species *GH3* genes changed obviously after allopolyploidization in *Brassica napus* [30]. During fruit development and maturation, *GH3* gene also showed different expression patterns. When IAA content was high, *GH3-1* gene was highly expressed in grape flowers and young fruits [31,32], whereas relatively low in roots and leaves [15]. *GH3.1/GH3-2* involved in the fruit ripening of grape berry [6,15]. *CcGH3* expression in shoots, buds, sepals, petals, and mature pericarp and placenta could be induced by auxin [17]. In longan fruit, *DlGH3.2* was believed to be involved in pericarp growth and fruit ripening [16]. A comprehensive expression profiling of auxin-related *GH3* genes in tomato, the expression levels of *SlGH3-1* and *SlGH3-2* increased at the early stage of fruit ripening, indicating that they may be involved in the initiation of fruit ripening [18]. *PpGH3-3* and *PpGH3-4* were significantly up-regulated in the mature stage, suggesting that they may be involved in the ripening and softening of peach fruit [33]. *PuGH3.1* plays an important role in the decrease of free IAA content in pear fruits, which is closely related to ethylene production and IAA content [7]. In our study, the expression of *AcGH3.1* shared high correlation with fruit postharvest ripening, and was induced by NAA and inhibited by 1-MCP. After transient silencing of its expression, fruit ripening could be delayed, suggesting that the *AcGH3.1* gene was involved in fruit ripening after harvest. This also provides a new idea for us to study the effect of IAA on the postharvest maturity of kiwifruit.

Further studies have shown that the expression of the *GH3* gene is affected by many other factors. Several endogenous and exogenous factors are currently known, including auxin (IAA), abscisic acid (ABA), ethylene, brassinolide (BL), gibberellin (GA), jasmonic acid (JA), salicylic acid (SA), light and biotic/abiotic stress can regulate the expression of *GH3* gene in plants [5,6,9,14,15,20,24,26,29,34,35]. The positive regulation of auxin is a common feature of group II *GH3* genes [5], and the presence of a core sequence (TGTCTC) of many auxin response elements (AuxRE) on the *GH3-1* gene promoter illustrates this problem [36]. GH3-1 expression in grapes can be induced by ABA or sucrose treatment, and the induction was amplified by the combination of the both, meanwhile, the presence of multiple ABA response elements (ABRE-like) in the *GH3-1* promoter region was predicted [15]. ABA and ethylene promote the expression of *DlGH3.2* during the ripening stage of longan fruits [16]. In peppers and pears, the expression level of *GH3* gene is strongly induced by ethylene, but is inhibited by 1-MCP [7,17]. Some *GH3* genes, such as *PBS3* (also known as *AtGH3.12*) and *AtGH3.5*, play key roles in regulating salicylic acid metabolism and inducing defense responses, suggesting that these genes can be regulated by other plant growth substances [35,37]. Transcriptional levels of *GH3.8* in rice can be rapidly induced by salicylic acid and jasmonic acid treatment [28]. In addition, *AtGH3.5* expression is induced by abiotic stresses such as cold, salt, drought and ABA [38], and cadmium was found to induce the up-regulation of some *GH3* genes in mustard [39]. Several types of transcription factors such as ARF (auxin response factor), bZIP (basic region-leucine zipper) and R2R3 type MYB (v-myb avian myeloblastosis viral oncogene homolog) have also been reported to effect the expression of the *GH3* gene in different plants [40,41,42]. When analyzing the *AcGH3.1* promoter sequence, we found that there are many cis-elements related to plant hormones in the promoter sequence, such as ABRE (abscisic acid responsive element), ERE-motif (ethylene response factor binding site), TCA-element (salicylic acid-responsive element.), and TGA-element (auxin-responsive element), which have been proved to play an important role in fruit ripening [43,44,45,46]. In addition, we found that the activity of the promoter of the *AcGH3.1* gene is affected by these exogenous hormones, which laid the foundation for our study of its upstream regulation. 

## 4. Materials and Methods

### 4.1. Plant Materials and Treatments

Kiwifruits (*A. chinensis*, Donghong) were collected at the commercially mature stage from the Institute of Kiwifruit Research in Fengxin County, Jiangxi Province, China. Fruit samples were divided into three groups; each treatment contained three biological replicates, each consisting of approximately 200 fruits. The first group was treated with 50 mg/L 1-naphthylacetic acid (NAA), the second group was treated with 1 μL/L 1-methylcyclopropene (1-MCP), and the third group was treated with air as a control. All fruits were enclosed in 0.02-mm thick polyethylene film bags to maintain relative humidity at nearly 95%, and then stored at 20 °C. At each sampling point, 3 replicates of 4 fruits each were collected from each treatment. The outer pericarp (without skin or seeds) of control fruits was separated at 0, 12, 24, 48 h, and 3, 6, 9, 12, 15, 18 d of storage, rapidly frozen in liquid nitrogen and kept at −80 °C until use.

### 4.2. Identification of the GH3 Gene from Kiwifruit Genome and Phylogenetic Analysis

Candidate genes encoding *GH3* were retrieved by BLASTP searching against the kiwifruit genome database (http://bioinfo.bti.cornell.edu/cgi-bin/kiwi/home.cgi), using tomato/*Arabidopsis* GH3 proteins as queries. Phylogenetic analysis was conducted using the ClustalW in software MEGA 7.0 (Arizona State University, Tempe, AZ, USA), and an unrooted phylogenetic tree of the *GH3* gene families was constructed using the software MEGA 7.0 [47]. Evolutionary history was inferred using the neighbor-joining method with 1000 replicates.

### 4.3. RNA Extraction and qRT-PCR

Total RNA was extracted using a plant RNA extraction kit (Huayueyang, Beijing, China). DNA contamination in the isolated RNA was digested by incubation with DNase I (Takara, Dalian, China) for 30 min at 37 °C before cDNA was synthesized; 1 μg RNA was used for cDNA synthesis using the Verso cDNA kit (Takara, Dalian, China); qRT-PCR was performed using a CFX96 Touch Real-time PCR (Polymerase Chain Reaction) instrument (Bio-RAD, Hercules, CA, USA) with 2 μL cDNA, 1 X TB Green Master Mix (5 μL) (Takara, Dalian, China), and 1 μM each of two gene-specific primers (Table S1), with a final volume of 10 μL in water. The thermal cycle scheme is as follows: 30 s at 95 °C, 40 cycles of 5 s at 95 °C, and 34 s at 60 °C. The *Actin* gene was used as internal reference. The relative expression of gene was calculated by the comparative cycle threshold method (△△*C*_t_). Three biological replicates and three technical replicates were performed to verify the accuracy of expression data.

### 4.4. AcGH3.1-Silenced Kiwifruit

A 486 bp fragment of the *AcGH3.1* gene was amplified by PCR from kiwifruit cDNA sources using gene specific primes GCTCTAGATTGAACCGCAATGGAACAGT (forward) and CGGGATCCTGATCCGATCCCAATTCTCAC (reverse) (*Xba I* and *BamH I* sites are underlined). The product of PCR was cloned into pTRV2 (posttranscriptional Tobacco Rattle Virus) at *Xba I* and *BamH I* restriction sites to form pTRV2-*AcGH3.1*. The purified plasmids of pTRV1, pTRV2, and pTRV2-*AcGH3.1* were transformed into *Agrobacterium tumefaciens* strain GV3101. Finally, *A. tumefacines* cells were resuspended with infiltration media (10 mM MgCl_2_, 10 mM MES (2-Morpholinoethanesulfonic Acid monohydrate), 200 μM AS (Acetosyringon)), adjusted to OD600 > 1.0. The liquid culture contained pTRV1 and pTRV2 (as a control) or pTRV1 and pTRV2-AcGH3.1 in a 1:1 ratio added and was cultured at 28 °C for 3 h. After this time, the liquid culture was infiltrated into the fruit pedicel approximately 7 d before harvest, after which, the *AcGH3.1*-silenced fruits were collected. Fruit firmness was measured as previously described [48], it was measured on opposite sides of each fruit after peel removal (1 mm thick), using a fruit texture analyzer (FTA, model GS, Güss Manufacturing Ltd., Strand, South Africa) with a 10-mm probe. Data were recorded as Newton (N) and fruit firmness was expressed as the mean of 15–20 fruits.

### 4.5. Cis-Elements Analysis

To investigate cis-elements in *AcGH3.1* gene promoter region, sequence of the 1287 kb region upstream of the start codons (ATG) was extracted from the kiwifruit genome database, and the gene promoter elements were predicted and analyzed using the PlantCARE online site (http://bioinformatics.psb.ugent.be/webtools/plantcare/html/) [49].

### 4.6. Promoter Activity Assay

The putative promoter region of AcGH3.1, a 1287 bp PCR fragment upstream of the start codon ATG, was amplified from kiwifruit genomic DNA using the primers AACTGCAGCTTTTATATATATATATATAT (forward) and CGGGATCCATATGAAGTGGTCCCCAATT (reverse) (*Pst I* and *BamH I* sites are underlined). The PCR product was digested with *Pst I* and *BamH I* was cloned in front of the *GUS* gene in the pCAMBIA1391 vector (promoter less vector), yielding the construct *proAcGH3.1-GUS*. The *AcGH3.1* promoter cloned in pCAMBIA1391 was transformed in *Agrobacterium tumefaciens* (GV3101). Then, *A. tumefaciens* containing the *proAcGH3.1-GUS* construct standing 3 h at room temperature. 500 μL *agrobacterium* was injected into the abaxial surface of the fully expanded tobacco leaves with a 1 mL syringe. After 3 days, different treatments were performed on the leaves. *Agrobacterium*-infected leaves were incubated at room temperature for 0, 6 or 12 h in petri dishes containing 10 μM abscisic acid (ABA), 10 μM ethephon, 10 Μm salicylic acid (SA) or 10 μM NAA, respectively. GUS staining was carried out as previously described [50].

## Figures and Tables

**Table 1:** The *GH3* gene family in kiwifruit.

Gene ^a^	ID	ORF Length (bp)	Chr ^b^	Position ^b^	Strand	Deduced Polypeptide ^c^ (kDa)		
						AA	MW	Pi
*AcGH3.1*	Achn117881	1437	27	6,340,994–6,342,430	+	478	54.29	5.48
*AcGH3.2*	Achn240911	1527	7	2,318,980–2,321,424	+	508	56.91	8.53
*AcGH3.3*	Achn261851	1500	10	3,736,109–3,738,789	−	499	56.03	7.50
*AcGH3.4*	Achn012711	1527	9	2,415,851–2,418,681	−	508	57.30	5.28
*AcGH3.5*	Achn333181	2163	23	7,342,673–7,356,855	+	720	81.26	5.71
*AcGH3.6*	Achn176981	1863	17	3,869,893-3,871,972	−	620	69.52	6.62
*AcGH3.7*	Achn379371	1728	Unknow	99,724,717–99,726,790	+	575	64.92	6.76
*AcGH3.8*	Achn169831	1812	6	131,509–134,022	+	603	68.24	5.34
*AcGH3.9*	Achn357511	1647	28	1,764,301–1,768,366	+	548	61.83	5.50
*AcGH3.10*	Achn042951	1584	23	12,408,214–12,411,765	+	527	59.88	5.38
*AcGH3.11*	Achn275921	1731	11	16,628,138–16,631,890	+	576	64.91	5.93
*AcGH3.12*	Achn283101	1743	8	7,649,263–7,652,985	–	580	65.40	5.50

^a^ Systematic designation given to kiwifruit *GH3* genes. ^b^ Chromosomal location of the *AcGH3* genes in the kiwifruit genome. ^c^ The theoretical isoelectric points (pI) and molecular weights (MW) of the deduced polypeptides were calculated using the ExPASy Compute pI/Mw tool (http://expasy.org/) [19].

**Table 2:** Pairwise comparison of amino acid identity (%) of 12 AcGH3 proteins.

Protein	AcGH3.1	AcGH3.2	AcGH3.3	AcGH3.4	AcGH3.5	AcGH3.6	AcGH3.7	AcGH3.8	AcGH3.9	AcGH3.10	AcGH3.11	AcGH3.12
AcGH3.1	100											
AcGH3.2	52.8	100										
AcGH3.3	52.1	93.5	100									
AcGH3.4	54.7	62.7	62.1	100								
AcGH3.5	39.3	46.5	46.5	41.3	100							
AcGH3.6	49.9	56.5	56.1	51.3	63.5	100						
AcGH3.7	49.4	69.8	59.3	52.6	64.3	90.2	100					
AcGH3.8	42.8	41.4	41.0	41.4	42.1	52.1	50.6	100				
AcGH3.9	47.1	38.0	40.3	39.6	35.9	48.3	44.9	50.7	100			
AcGH3.10	21.2	20.5	20.5	21.3	23.9	27.2	26.3	27.8	25.7	100		
AcGH3.11	31.3	30.3	31.0	32.3	29.0	36.3	35.3	36.7	34.5	43.9	100	
AcGH3.12	30.9	30.1	30.6	31.7	28.5	35.6	34.0	36.9	34.7	45.1	93.2	100

**Table 3:** Cis-elements were predicted in the promoter regions of *AcGH3.1* related to hormone and abiotic stress in the kiwifruit.

Cis-Element	Sequence	Function of Site	Copies
ABRE	ACGTG	Abscisic acid-responsive element	3
G-Box	CACGTT	Light-responsive element	3
ERE	ATTTTAAA	ERF (ethylene response factor) binding site	4
TCA-element	CCATCTTTTT	Salicylic acid-responsive element	1
TGA-element	AACGAC	Auxin-responsive element	1
MBS ^1^	CAACTG	Drought-inducibility	1

^1^ MYB binding site.

## Supplementary Materials

The following are available online at https://www.mdpi.com/2223-7747/8/11/473/s1, Table S1: Primers for qRT-PCR.
