# Supplementary material for: Identification and Expression Analysis of Gretchen Hagen 3 (GH3) in Kiwifruit (Actinidia chinensis) During Postharvest Process
Source: Plants (Basel). 2019 Nov 6;8(11):473. doi: 10.3390/plants8110473 (PMC6918289; doi:10.3390/plants8110473)
Supplement: Supplementary file 1 [file plants-08-00473-s001.pdf]

Table S1. Primers for qRT-PCR.

Primer name	Forward sequence	Reverse sequence
AcGH3.1	TACCTCCGCCGATTCAAACTC	GATGGGATGTGCCGACAAGAT
AcGH3.2	GGGGCTCTATTTCCTCTTCAT	TCGTTCGGGCTCGTGTAT
AcGH3.3	AGGGTCTGTACTTCCTGTTCG	TGTAGACGTTGTAGGGATCGTAG
AcGH3.4	AAGGAGTGCGAAGGTGAGAAC	AAGTAGTCGAGGGTCGGGAT
AcGH3.5	TCAATTCCTCCTTCGGTCTAT	AACGTCCCAGCTTCAACAA
AcGH3.6	CATCAAATCGGAGGCTAAG	GTATGAGTCAGGGCACAAAA
AcGH3.7	ATCTCCCATCCTCTGTTCCC	GCAATGACCTCCTCCCTATC
AcGH3.8	AGAAACCGTCCCTTCAACCC	GCACCAACCCTAAGAACCTC
AcGH3.9	TTCCTCCGAGTGTTACTTTGGT	CCTTAGCCACCTCCTCTTCTTC
AcGH3.10	CCCAAGAATCACCATACCCAG	CGGAACCAATCCAAACCAG
AcGH3.11	AGTTCATCTACAGCAGCAAG	TCAGGACCAAATATCACTTC
AcGH3.12	TCATCTACAGCAGCAAGCAG	GGAAGTCAGGACCAAATATCAC
AcActin	TGGAATGGAAGCTGCAGGA	CACCACTGAGCACAATGTTGC
